# Connect dispatch centers for call handling improves performance

**DOI:** 10.1186/s13049-019-0601-y

**Published:** 2019-02-20

**Authors:** Yann Penverne, Michel Terré, François Javaudin, Joël Jenvrin, Frédéric Berthier, Julien Labady, Brice Leclere, Emmanuel Montassier

**Affiliations:** 1 Department of Emergency Medicine, SAMU44, CHU Nantes, Nantes, France; 2 Conservatoire National des Arts et Métiers CEDRIC, 292 rue Saint Martin, 75003 Paris, France; 3 MiHAR lab, Université de Nantes, 44000 Nantes, France; 4 Department of Medical Evaluation and Epidemiology, Nantes University Hospital, 85, rue St Jacques, 44093 Nantes Cedex 1, France

**Keywords:** Emergency medical dispatch, Emergency medical services, Virtualization

## Abstract

The aim of this Letter to the Editor was to report a strategy to reduce time waiting for emergency calls in a dispatch center, in line with a recently published article that reviewed evidence for medical dispatching systems to accurately dispatch Emergency medical Services. Here, we tested the effect of a connected distribution of calls, where a call is allocated to the first available resource among a pooled group of telecommunicators from several dispatch centers. We found that connect dispatch centers improve dispatch center performance, especially during an overloaded period. It could be leveraged to handle emergency calls without delay and to appropriately dispatch Emergency Medical Services.

To the Editor:

We have read with great interest the article, “The accuracy of medical dispatch - a systematic review” by Bohm and Kurland [1]. The authors reviewed evidence for medical dispatching systems to accurately dispatch Emergency medical Services (EMS), arguing that the challenge is to dispatch EMS appropriately with limited resources. Indeed, dispatch centers receive an increasing number of emergency calls each year, and calls are traditionally routed to the local dispatch center. However, temporal variations of the daily activity emphasize the importance of scheduling process [2–4]. Allocation of additional resources to an activity peak could be a solution. However, impact of such strategy is limited as telecommunicators workforce is constrained and not easily mobilizable for a brief work shift corresponding to the activity peak. To date, collaboration between dispatch centers for call handling process does not exist. Virtual phone system for call distribution consists in distributing calls to connected resources from different geographic locations. Automatic call distribution is achieved through an advanced telephonic system, which dynamically balances the workload and allocates calls to available telecommunicator. This operational model enables rapid contact through the pooling of available resources [5]. Thus, the objective of our work was to study the effect of a connected distribution of calls on dispatch center performance.

We compared two models of call distribution: i) monocentric call distribution to call takers of the corresponding geographic area (i.e., calls routed to the local dispatch center) and, ii) virtual phone system distribution to a group of dispatchers physically distributed in several dispatch centers and connected to the same information system, so that the calls are distributed to the resource immediately available in this group. We set the number of dispatchers in each site at five. Based on our previous work, we considered a nominal situation in which the activity observed on a weekday was on average 39 calls per hour, with an average duration of a call of 106 s [3]. This activity was the basic traffic. We defined the following telephone flows to reflect temporal variation of the activity of the dispatch center: i) Loaded traffic: in busy hours, the traffic is doubled compared to the basic traffic, the average calls per hour is equal to 78, which leads to a rate of new calls called λ, equal to 78/3600 calls per second. The traffic is measured in Erlang, a unit of measurement of the telephone traffic that represents the rate of occupation of a resource [6]. A caller who is continuously on call throughout the day would therefore occupy a telephone resource 100% of the time and thus generate a 1-Erlang traffic. Keeping the average call duration, rated 1/μ, equal to 106 s, we get a traffic A = λ/μ, which gives A = 2297 Erlangs; ii) Traffic T75: in periods of high activity, we apply an increase of 75% of the traffic hours loaded, corresponding to 4019 Erlangs; iii) Traffic T100: in periods of high activity, we also apply a 100% increase in the traffic hours loaded, corresponding to 4593 Erlangs.

In periods of high activity corresponding to 75% of the traffic hours loaded, with a monocentric call distribution (i.e., calls routed to the local dispatch center), the probability to be put on hold was 56.2%, with an average waiting time of 61 s. The probabilities of waiting less than 20 s and 6 s were 53.3 and 46.9%, respectively. In the virtual phone system model, with a call distribution connecting six dispatch centers, the probability to be put on hold was 18.1%, with an average waiting time of 4 s. The probabilities of waiting less than 20 s and 6 s were 94 and 87%, respectively. To achieve the same results with a monocentric call distribution, number of telecommunicators should be increased from 5 to 8 (i.e., 80% increase). Moreover, when eleven dispatch centers were connected, the probability to be put on hold was 7.9%, with an average waiting time of 1 s. In this case, the probabilities of waiting less than 20 s and 6 s were 99 and 95.7%, respectively. We also tested our model in periods of high activity corresponding to 100% of the traffic hours loaded. With a monocentric call distribution model, the probability to be put on hold was 80.5%, with an average waiting time of 210 s. The probabilities of waiting less than 20 s and 6 s were 25.5 and 21.4%, respectively. In the virtual phone system model, with a call distribution including twenty dispatch centers, the probability to be put on hold was 30.4%, with an average waiting time of 4 s. The probabilities of waiting less than 20 s and 6 s were 93 and 81% respectively. To achieve the same results with a monocentric call distribution, number of telecommunicators should be increased from 5 to 9 (i.e., 90% increase).

Here, we found that connecting dispatch centers can help to improve time waiting for emergency calls. Our results support that dispatch center virtualization could be applied in case of activity peak, by determining the available telecommunicator in a large panel of call takers connected from different geographic locations. Thus, virtual phone system for call distribution can improve dispatch center accessibility, especially during an overloaded period. It could be leveraged to handle emergency calls without delay. Programs of dispatch center modernization should discuss the implementation of such model of call distribution. Collaborative projects between dispatch centers should be created to promote rapid access to a telecommunicator, to evaluate whether emergency medical services are needed and with which priority the resource needs to be dispatched [7].

## Abbreviation

EMS: emergency medical services
